# A systematic method to identify modulation of transcriptional regulation via chromatin activity reveals regulatory network during mESC differentiation

**DOI:** 10.1038/srep22656

**Published:** 2016-03-07

**Authors:** Zhana Duren, Yong Wang

**Affiliations:** 1 Academy of Mathematics and Systems Science, National Center for Mathematics and Interdisciplinary Sciences, Chinese Academy of Sciences, Beijing 100190, China

## Abstract

Chromatin regulators (CRs) are crucial for connecting the chromatin level and transcriptome level by modulating chromatin structures, establishing, and maintaining epigenetic modifications. We present a systematic method to identify MOdulation of transcriptional regulation via CHromatin Activity (MOCHA) from gene expression data and demonstrate its advantage in associating CRs to their chromatin localization and understand CRs’ function. We first re-construct the CRs modulation network by integrating the correlation and conditional correlation concepts. Then we quantify the chromatin activity as hidden variable in network by integrating the upstream and downstream information. We applied MOCHA to systematically explore the interplay of CRs, TFs, and target genes in mouse embryonic stem cells (ESC). As a result, MOCHA identified 420 chromatin regulators with modulation preference, including Pou5f1 and Eed. We found that BAF complex, NuRD complex, and polycomb-group proteins, regulate the delicate balance between pluripotency and differentiation by modulating key TFs including Klf4, Tcf3, and Max; NuRD complex members Mbd3 and Hdac1 may modulate Klf4 to achieve its dual functional roles in pluripotent and differentiation stages;Imprinted gene H19 and Igf2 are modulated by DNA methylation, histone acetylation, and insulator CTCF. Finally, we analyzed CR’s combinational modulation pattern by constructing a CR-CR interaction network.

Embryonic stem cells (ESCs) possess the unique property of pluripotency, which is the ability to develop into any cell lineages of the organism. Consequently, a detailed knowledge of the mechanisms governing lineage specific differentiation in ESCs is necessary to realize the potential of these cells. We have the rough picture that epigenetic and genetic factors interplay actively and together lead to phenotype change in the differentiation process. Specially, the chromatin structure is changing dramatically along the stem cell differentiation and highly cell-type specific^1^,^2^. Recent studies indicate that genome-wide transcriptional levels are highly correlated with chromatin state switches^3^,^4^. In addition, the chromatin state switches are connected with the fine-tuning gene expression patterns in developmental stage-specific manner^5^,^6^. In combination, these studies suggest that chromatin state plays an important role in transcriptional regulation during stem cell differentiation.

Similar to the thousands of transcription factors’ (TFs) regulation in gene expression level, the genome also encodes hundreds of chromatin regulators (CRs) to modulate the chromatin level by adding (writer), removing (eraser), or binding (reader) modifications^7^. It’s known that CRs play important roles in many biological processes, for example, mutations in CR genes revealed by cancer genome projects suggest its connections with normal physiology and disease^8^. One straightforward way to understand CRs’ function is tracking the localization of CRs in chromatin by its target loci. Robert F. *et al.*^9^ explored the genome-wide occupancy of four different chromatin regulators encoded in *Saccharomyces cerevisiae*. However, systematic localization of mammalian CRs has been proven challenging. Recently, Oren Ram *et al.*^7^ proposed a general methodology to map CRs in mammalian cells and demonstrated the usefulness of studying the localization of CRs in K562 cells and human embryonic stem cells. The resulting datasets provide a comprehensive view of the associations between CRs and histone modification states. Thanks to those technologies, massive data have been accumulated. For example, Qixuan Wang *et al.*^10^ collected all publicly available ChIP-Seq data on CRs in human and mouse into four cohorts: the reader, writer, eraser, and remodeler.

In general, at least three processes control the assembly and regulation of the chromatin: DNA methylation, histone modification, and ATP-dependent chromatin remodeling enzymes^11^. Furthermore, an open chromatin state correlates with a globally permissive transcriptional state, and has been proposed that this state allows transcriptional programs to switch rapidly upon induction of differentiation^12^. CRs are important in those chromosome maintenance procedures. However, the location of CRs in chromatin is only the first step to understand CRs’ function *in vivo*. Ultimately we need to dig into the transcriptional level to pursuit their fundamental roles in regulating gene expression. Here, we aim to study how the transcriptional regulations are modulated by the chromatin states and further CRs. Toward this end, we propose a systematical method for identifying MOdulation of transcriptional regulation via CHromatin Activity (MOCHA). Our method, MOCHA, utilizes the gene expression data to study the relationships among CRs, TFs, and target genes (TGs) by integrating the correlation and conditional correlation concepts.

Specifically, our computational model integrates transcriptional regulation information, modulation of chromatin information, and phenotype information to score a given triplet consist of chromatin regulator, transcriptional factor, and target gene. By further investigating the location of target genes in chromatin, we detect the chromatin modulation of transcriptional regulation via a given genomic region. Inside each region, we apply a Gaussian Bayesian model to infer the chromatin activity by integrating the *cis* and *trans* regulations. At last, we construct a network for chromatin regulator cooperation by their sharing genomic regions and systemically characterize their biological functions.

We applied this approach to detect the chromatin modulation of transcriptional regulation in the early stage of mouse embryonic stem cell differentiation. We found both known and novel regulations for the important factors during this process. This demonstrates the efficiency of our new method and provides important insights for the chromatin regulators.

## Results

### MOCHA integrates genomic data to reveal chromatin regulator’s function

**Figure 1:**
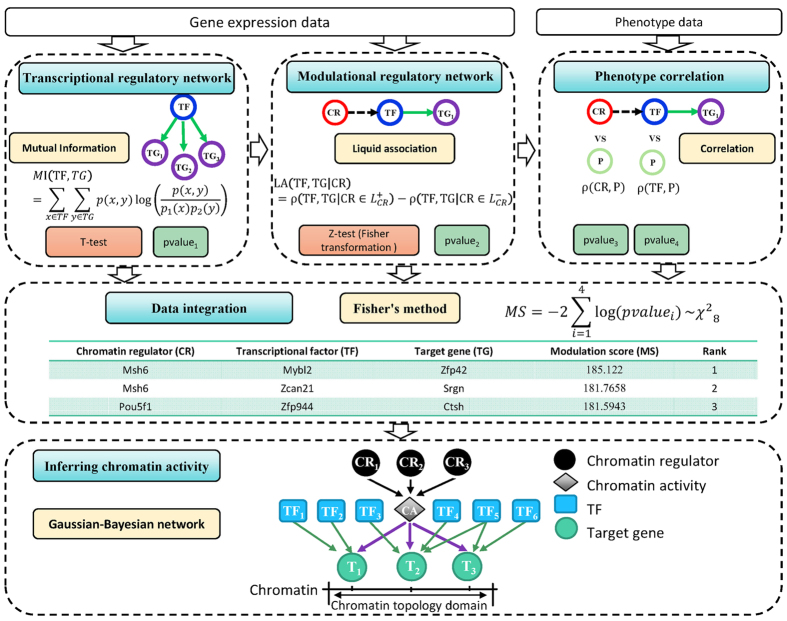
Workflow of MOCHA. MOCHA is a method to identify CRs’ modulation role in transcriptional regulation. MOCHA takes gene expression data and phenotype data as input. It will output the chromatin activities in each genomic region and their corresponding chromatin regulators. All the triplets composed of chromatin regulator (CR), transcriptional factor (TF), target gene (TG) (modulation on a single target gene) are ranked by an integrative modulation score. Given a triplet, it computes modulation score by integrating the transcriptional regulation strength, interaction of transcriptional regulation and chromatin regulator, consistence of chromatin regulator expression and phenotype, and consistence of TF expression and phenotype. All the four lines of evidence can be evaluated by p-values and further combined into a single modulation score by Fisher’s method. We then rank all the triplets by their modulation scores. At last, the significant triplets are integrated into a graphical model according to their target gene’s genomic location to infer the chromatin activity of each chromatin region.

We propose MOCHA as a novel method to reveal modulation of transcriptional regulation via chromatin activity from gene expression data (Fig. 1). MOCHA identifies CRs’ modulation role on transcriptional regulation via a single target gene or a genomic region. MOCHA takes gene expression data and phenotype data as input and ranks all the triplets of chromatin regulator (CR)-transcriptional factor (TF)-target gene (TG) (modulation on a single target gene) by a well-defined modulation score. It will output the chromatin activities in each genomic region and their corresponding chromatin regulators (modulation of a genomic region). Given a triplet composed of a chromatin regulator and a pair of transcriptional regulation (TF target gene pair), we compute the modulation score by integrating four pieces of evidence: the transcriptional regulation strength, interaction of transcriptional regulation and chromatin regulator, consistence of chromatin regulator expression and phenotype, and consistence of TF expression and phenotype.

**Figure 2:**
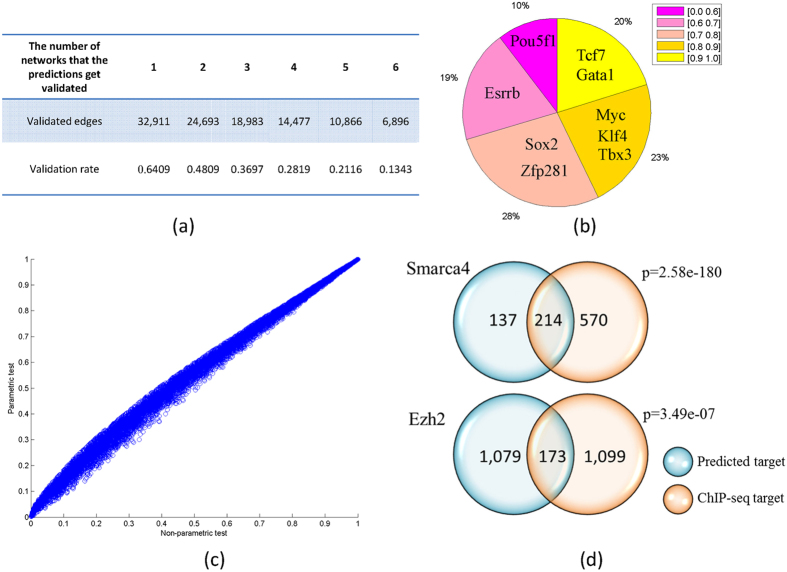
Validation of MOCHA predictions. (**a**) The inferred transcriptional regulation network is validated by six DNase-seq data derived networks. The smallest DNase-seq data derived network has 781,636 edges and the biggest one has 989,520 edges. (**b**) Evaluation of the target gene prediction for each TF by mutual information. AUC score for each TF is computed and shown in pie graph. Each color means a bin for AUC score, for example [0, 0.6], [0.6, 0.7], [0.7, 0.8], [0.8, 0.9] and [0.9, 1.0].The percentage represents the percent of TFs in each AUC bin. (**c**) Comparison of liquid association p-values computed by our method and non-parametric permutation test. (**d**) Comparison of predicted CR target genes and CRs’ target genes measured by ChIP-seq. We apply our method to predict target genes on polycomb complex member Ezh2 and BAF complex member Smarca4 (BAF190) which have ChIP-seq data to compare in mESC. As a result, 214 out of 351 and 173 out of 1,252 target genes are validated by the Smarca4 and Ezh2 ChIP-seq data respectively. Both of these two overlapping are significant by hypergeometrical test.

Mutual information is used to infer the transcriptional regulatory network (see Methods and Materials). We inferred 237,450 transcriptional regulation relationships for 943 TFs by the cutoff of significant level 0.005 (Supplementary dataset 2). We compared with public DNase-seq data derived networks^13^ and finds that 64% interactions in our network are supported by DNase-seq footprint evidence (Fig. 2(a,b)). For 71% of the TFs, their target genes can be predicted by mutual information with high accuracy (AUC > 0.7).

Then we propose a fast approximate liquid association^14^, an elaborate method for the genome-wide co-expression dynamics analysis, to detect the strength and pattern of associations between two gene profiles modulated by chromatin remodeling factors, chromatin modifying enzymes, and some epigenetic factors (Fig. 2(c)). To validate the results, we compare the predicted CR target genes with CR’s available ChIP-seq data. We apply our method to predict target genes on polycomb complex member Ezh2^15^,^16^,^17^ and BAF complex member Smarca4 (BAF190)^18^ which have ChIP-seq data to compare in mESC^10^. As a result, 214 out of 351 and 173 out of 1252 target genes are validated by the Smarca4 and Ezh2 ChIP-seq data respectively. Both of these two overlapping are significant (Fig. 2(d)). These examples demonstrate MOCHA’s advantage to utilize only gene expression data to predict CR’s genomic localization in chromatin. Given the difficulty to generate ChIP-seq data for CRs experimentally, MOCHA offers one simple and cheap way to provide rich information for CR’s function.

To further narrow down the number of CR-TF-TG triplets, we introduce a pluripotency index to incorporate the phenotype information of the samples. The pluripotency index is defined as the mean expression of 16 core pluripotency genes (Pou5f1, Sox2, Nanog, Klf4, Myc, Tcf3, Esrrb, Zfp42, Nr0b1, Stat3, Sall4, Phc1, Klf2, Rest, Zfp281, and Tbx3). For each time point we can calculate this pluripotent index and use it to monitor the phenotype change during the differentiation (see Methods and Materials for details). Given a modulation score for a given triplet, we apply Fisher’s combined probability test to integrate four p-values: mutual information p-value, liquid association p-value, the p-value of correlation between CR and phenotype, the p-value of correlation between TF and phenotype (Fig. 1). In this way, all the triplets have a modulation score (chi-square score) and an integrative p-value (Supplementary Figure 1). Finally we integrate these triplets into high level structures to a graphical model and infer the chromatin regulators and chromatin activity of each chromatin region (see Methods and Materials).

### The chromatin regulators modulate significantly more transcriptional regulations

**Figure 3:**
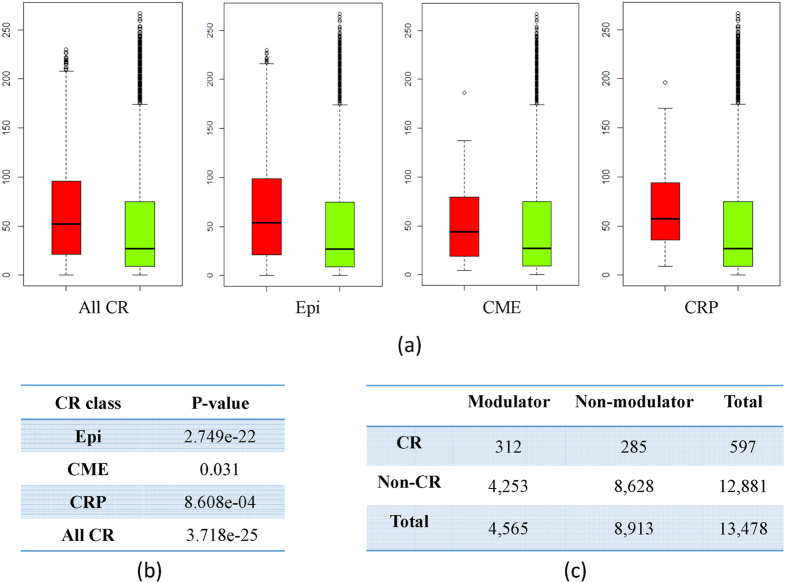
The chromatin regulators tend to modulate more transcriptional regulations. (**a,b**) Comparing CRs (red) with non-CR genes (blue) by the number of the TFs modulated and its corresponding p-values. Epi, CME, and CRP are the three CR categories. (**c**) By a cutoff of 50 (a modulator is a gene modulating more than 50 TFs), the CR genes are more likely to function as modulators than non-CR genes (Chi-square test, p-value < 1.00e-16).

We screened the whole genome to see whether MOCHA can detect the modulation of the transcriptional regulation from gene expression data. All the regulations modulated by each gene (CR or non-CR) are ranked. We compared CRs and non-CRs by the number of regulations they modulate. The results show that chromatin regulators tend to modulate significantly more transcriptional regulations than non-CR genes (t-test, p-value < 3.72e-25). Furthermore, we looked into the three types of chromatin regulators and compared the modulation degree of epigenetic factors, chromatin modifying enzymes, and chromatin remodeling proteins with non-CR genes respectively. The results are consistent that all three categories of CRs tend to modulate more transcriptional regulations (Fig. 3). These results indicate that chromatin factors play a broad role by acting on chromatin. In addition to the direct ChIP-seq data validation, it also demonstrates that our method can detect the modulations from gene expression data successfully. Interestingly, we find that the epigenetic factors modulate a relative large number of transcriptional regulations than the other two CR classes. This result is consistent with the knowledge that the epigenetics landscape has dramatic influence in stem cell differentiation stage^19^.

### Topological analysis of the modulation network reveals key regulators of stem cell development

**Figure 4:**
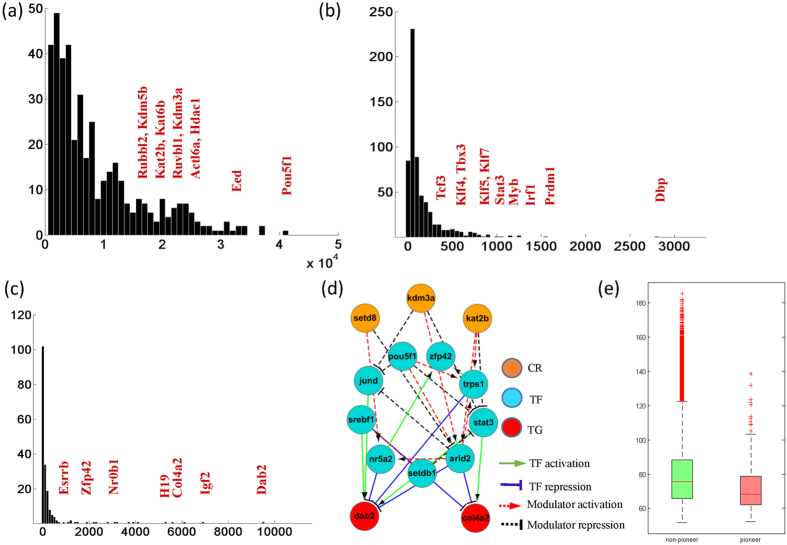
Topology analysis of the modulation network. (**a**) Histogram of the chromatin regulation scores for CRs. (**b**) Histogram of the number of TFs in the triplets. (**c**) Histogram of the number of target genes in the triplets. (**d**) A module in modulation network. (**e**) Comparison of in-degree of pioneer TFs and non-pioneer TFs in modulation network.

By running on the gene expression dataset for mESC differentiation, MOCHA outputs a set of triplets with 420 chromatin regulators, 612 transcriptional factors, 195 target gene, 3,185 transcriptional regulation edges, 35,426 modulation edges, and 84,184 modulation-transcriptional regulation triplets (Supplementary dataset 3). The chromatin regulation score of the chromatin regulators reflects the importance of this CR during stem cell differentiation. Figure 4(a) plots the histogram of the chromatin regulation score. We find that the chromatin regulator with the highest score is the well-known stem cell marker Pou5f1. We also find that Eed, a member of polycomb complex PCR2, has a high chromatin regulation score. These results indicate that polycomb group plays an essential role in stem cell differentiation, which is consistent with the existing findings^20^. In addition, some important chromatin regulators such as the SWI/SNF family member Actl6a, the CHD family protein Hdac1, INO80 family member Ruvbl1, and Rrubl2, K-demethylases family enzymes Kdm3a and Kdm5b, K-acetyltransferase family enzymes Kat6b and Kat2b have high chromatin regulation scores.

Key TFs in developmental stage are detected in the triplets too. Figure 4(b) shows the histogram of the number of TFs involved in the triplets. Some pluripotent factors such as Tcf3, Klf4, Klf5, Klf7, Stat3, Tbx3, and Myb have high degrees in the network. However, the top three TFs with highest degree are Dbp, Prdm1, and Irf1. Recent study demonstrates that a critical requirement for Irf1 in the generation of Tr1 cells both *in vitro* and *in vivo*, indicating that Irf1 is a key transcriptional regulator of Tr1 differentiation^21^. Prdm1 is reported to be a master regulator for plasma cell differentiation^22^ and Dbp may be involved in the proliferation of the hepatocytes.

Differentiation associated genes are present in the modulation network frequently. These genes are regulated by different TFs and these regulations are also modulated by different chromatin regulators. For example, Dab2 is a hub gene in the modulation network and it is regulated by Stat3, Aaid2, and Nr5a2. These regulations are modulated by Kdm3a, Kat2b, and Setd8 (Fig. 4(c,d)). Differentiation associated factors Igf2, Col4a2, and H19 are regulated by many TFs and have high degrees in the network. Interestingly, pluripotent factors Nr0b1, Zfp42, and Esrrb are also regulated by many TFs, which indicate that these factors’ functions may be switched along with the cellular state transformation (Fig. 4(c)).

We piled up all the triplets into a network, named modulation network. It consists of two classes of edges: transcriptional regulation edges (from TF to target gene) and modulation edges (from chromatin regulator to TF). We applied MCODE method^23^ to detect the densely connected modules in the modulation network. As a result, we found 11 modules and one example is shown in Fig. 4(d). This module contains three chromatin regulators Kdm3a, Kat2b, and Setd8, nine TFs including pluripotency factor Pou5f1, Zfp42, and Stat3, and two target genes Dab2 and Col4a2. All the three chromatin regulators are chromatin modifying enzymes and they are members of three different families: K-demethylase family, K-acetyltransferase family, and K-methyltransferase family. These results indicate that chromatin modifying enzymes may cooperate with key TFs to promote differentiation.

In the modulation network, we explore the function of pioneer TFs, a subset of TFs that occupy previously closed chromatin and, once bound, allow other TFs to bind nearby^13^. We compared the in-degree of pioneer TFs and with non-pioneer TFs. The results show that non-pioneer TFs tend to be heavily modulated by more CRs than pioneer TFs (Fig. 4(e)). This result may indicate that pioneer TFs’ regulation on downstream genes doesn’t heavily reply on the help of chromatin regulators, which is very different with non-pioneer TFs. This fact is consistent with the knowledge that pioneer TFs tend to binding to closed region directly and play a key role in shaping the chromatin landscape^13^.

### NuRD complex modulates the dual function of Klf4 by histone acetylation

**Figure 5:**
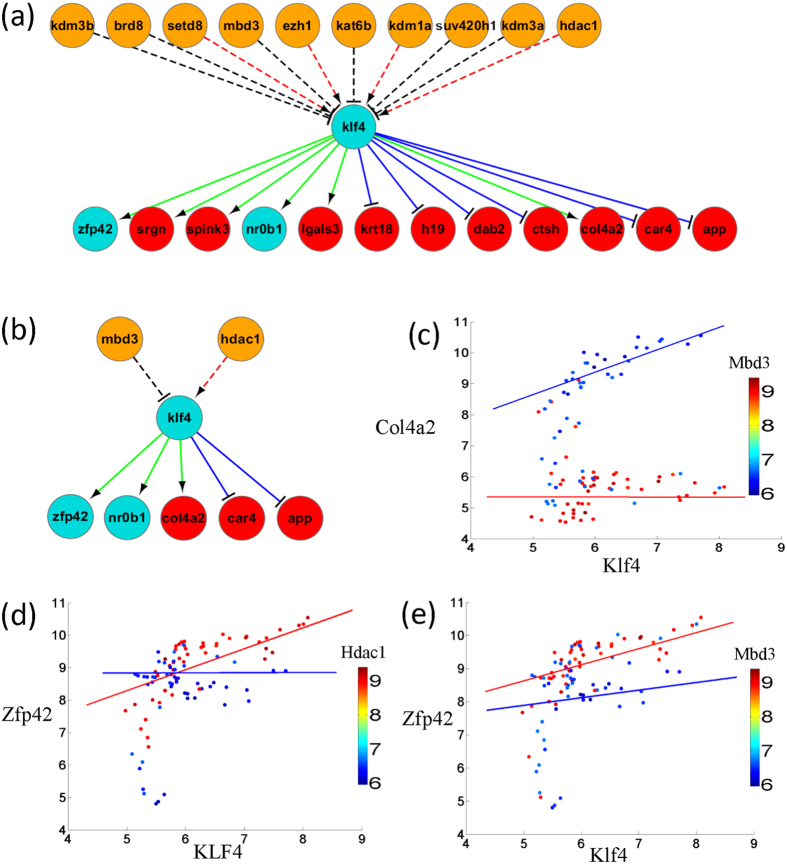
The NuRD complex modulates the regulation between Klf4 and its target genes by histone acetylation. (**a**) Subnetwork of Klf4 in modulation network. (**b**) Subnetwork of Klf4 with chromatin regulator restricting on NuRD complex. (**c**) Expression pattern of Klf4 and Col4a2 changes dynamically conditioning on the expression of chromatin regulator Mbd3. (**d**) Expression pattern of Klf4 and Zfp42 changes dynamically conditioning on the expression of chromatin regulator Hdac1. (**e**) Expression pattern of Klf4 and Zfp42 changes dynamically conditioning on the expression of chromatin regulator Mbd3.

We analyze the modulation subnetwork of Klf4. We find that Klf4 regulates some important pluripotent factors including Zfp42 and Nrob1. These regulations are modulated by CHD family NuRD complex, K-methyltransferase family, and K-acetyltransferase family members (Fig. 5(a)). Klf4 regulates Zfp42, Col4a2, Krt18, and Lgals3, which are validated by ChIP-seq and ChIP-chip data^24^. Most of the remaining targets except Nrob1 are validated by the transcriptional regulation network derived from DNase-seq data^13^. Interestingly, the regulation between Klf4 and Zfp42 dynamically changes along the differentiation stage, which is also captured by the DNase-seq derived network. These results suggest that the subnetwork around Klf4 is in high precision. In addition, dynamic regulation on target gene may indicate a novel mechanism of chromatin regulation on Klf4. To explore the dual function of Klf4, we analyzed the upstream chromatin regulators of Klf4. The regulation between Klf4 and Zfp42 is modulated by the chromatin regulator methyl-CpG binding domain protein 3 (Mbd3) and histone deacetylase 1 (Hdac1) (Fig. 5(b)). Both Mbd3 and Hdac1 are the members of the CHD family NuRD complex, which is a multisubunit complex containing nucleosome remodeling and histone deacetylase activities. Down regulation of this complex is required for the efficient reprogramming^25^. In addition, both Mbd3 and Hdac1 are reported to be associated with stem cell pluripotency. Mbd3 is necessary to maintain the pluripotency of mouse embryonic stem cell^26^ and have direct interactions with Oct4, Sox2, Klf4, and C-myc^27^. Hdac1 may control stem cell differentiation^28^ and to repress cell proliferation through interplay and modulation of Klf4 expression^29^. These evidences indicate that NuRD complex member Mbd3 and Hdac1 modulate the regulation of Klf4 and explain its dual functional roles in pluripotent and differentiation stages. Furthermore, switch of different roles in different stages is also observed by histone deacetylase. Klf4 regulates Zfp42 and doesn’t regulate Col4a2 in low acetylation state. Oppositely, Klf4 regulates Col4a2 and doesn’t regulate Zfp42 in high acetylation state (Fig. 5(c–e)).

Dual function of pluripotent factors plays a key role in stem cell differentiation. Besides Klf4, there are also some genes performing dual function by regulating the delicate balance between pluripotency and differentiation, such as Tcf3, Max, and Myb. Tcf3 regulates Igf2, Max regulates Dab2 and, Myb regulates Nr0b1. These three pairs of regulation are present in pluripotent stage and differentiated stage with different co-expression patterns (Supplementary Figure 3).

### Modulation of transcriptional regulation via genomic regions

Starting from the identified triplets, we further apply the Gaussian Bayesian network model to identify the regulatory modules to constrain the target genes in a genomic region. We hypothesize that chromatin regulators actually regulate target genes by physically affecting the chromatin state of a genomic region. To validate the accuracy of the CR target region in chromatin, we compare the CR predicted target region with the CR ChIP-seq peaks on polycomb complex member Ezh2 and BAF complex member Smarca4. As a result, both these two CRs’ predicted target region tends to overlap with ChIP-seq peaks (Supplementary Figure 2). Then we study the CRs’ localization on genomic regions. The results show that DNA methyltransferases Dnmt3a, Dnmt3b, and Dnmt3l globally regulate many chromatin regions. This is consistent with the present study which find that DNA methyltransferases are essential for the mammalian development^30^. The chromatin remodeling BAF complex member Actb and CHD family member Rbbp7, which is a member of ISWI family, also regulate many chromatin regions.

**Figure 6:**
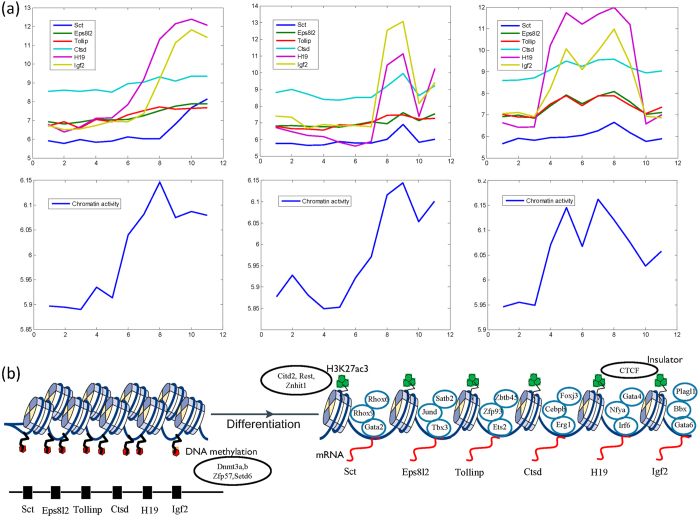
Modulation of transcriptional regulations in genomic regions. (**a**) Gene expression and inferred chromatin activity of genomic regions in three independent datasets. (**b**) Mechanism of chromatin activation by epigenetic regulation during stem cell differentiation. Genes in black circle are CRs and genes in blue circle are TFs.

Target genes are often regulated by the cis-regulatory elements and trans-regulatory elements. Chromatin activity is a concept to quantify the state of a cis-regulatory element which reflects the chromatin structure, accessibility, and modifications. Sct, Eps8l2, Tollip, Ctsd, H19, and Igf2 are located in genomic region from 141,278,331 to 142,666,816 in chromosome 7. These genes are regulated by the same cis-regulatory element, whose state can be quantified by chromatin activity. Their gene expression patterns are quite similar in three independent datasets (Fig. 6(a)). Different expression patterns are explained by different trans-regulatory elements. We apply our model to infer the activity of the cis-regulatory element and the results show that the inferred chromatin activity is consistent with the common expression pattern of these target genes. This region may have a highly compacted structure and remain available for the induction of developmental programs. Transcriptional factors are trans-regulatory elements and regulate the genes under the condition that the chromatin is active. Therefore, the chromatin regulators activate this region gradually during cell differentiation. Transcriptional factors activate the expression of target genes.

From the Gaussian Bayesian network we can infer the key chromatin regulators which potentially activate this region. Mainly two groups of chromatin regulators play key roles in this process (Fig. 6(b)). The first one is DNA methylation associated factors, such as Dnmt3a, Dnmt3b, Zfp57, and Setd6. These regulators are highly expressed in pluripotent state and descend along the differentiation. Oppositely, the second regulatory group, histone acetylation associated factor Citd2, Rest, and Znhit1 are lowly expressed in pluripotent state and increases along the differentiation. These results suggest that this region has high DNA methylation level in pluripotent state and thus the target genes are lowly expressed. Furthermore, high histone acetylation level in differentiated state activates the expression of the target genes. For example, H19 and Igf2 are reciprocally imprinted and play key roles in embryonic development^31^. Interestingly, DNA methylation and histone acetylation are essential for gene imprinting can be found in our result. This is consistent with the known results^32^. More interestingly, another important chromatin regulator of H19 and Igf2 is the insulator CTCF, which is known to be essential for enhancer blocking and is the main factor involved in the reciprocally imprinting of H19 and Igf2^33^.

### Combinational modulation of chromatin regulators

**Figure 7:**
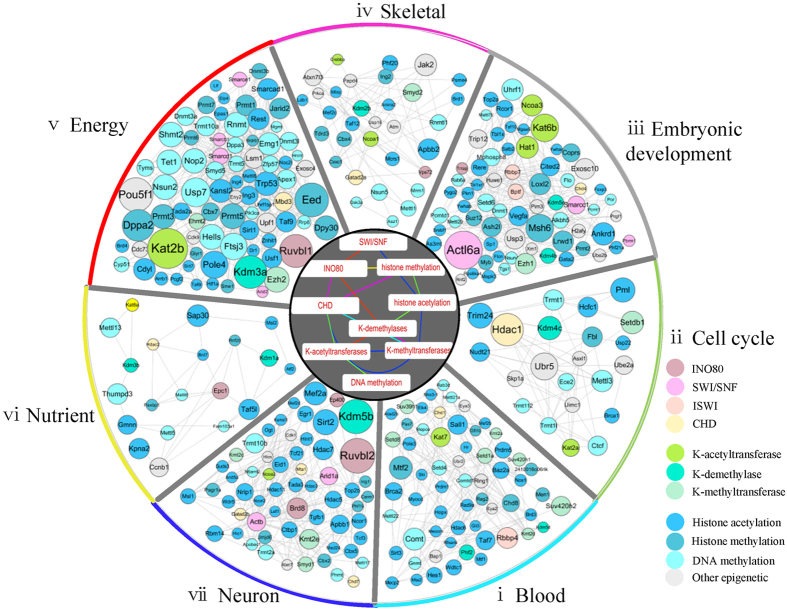
CR network reveals combinational modulation of chromatin regulators. Subnetwork in each sector is identified as a module. The node size is proportional to the CR’s chromatin regulation score. The network in the center of the circle is a network among CR-classes. An edge between two CR-classes represents that these two CR-classes are connected closely in a module, which is indicated by edge color.

To detect the combinational modulation pattern of the chromatin regulators, we construct a CR-CR interaction network by counting their shared genomic regions inferred from the Gaussian Bayesian model. We apply Fisher’s exact test to evaluate the significance of the number of shared genomic regions. On a significance level 0.005, we get a CR-CR interaction network which contains 342 CRs and 1,475 interactions (Supplementary dataset 4). To explore the detail of this network, we apply Newman’s fast algorithm^34^ to partition the network into 7 densely connected modules (Fig. 7).

At first, we test the preference of the CRs in each module. We find that different CR classes are enriched in different modules. For example, K-methyltransferase family CRs are enriched in module i; ISWI family CRs are enriched in module iii; DNA methylation associated CRs are enriched in module v; K-demethylase family CRs are enriched in module vi; INO80 family CRs and histone acetylation associated CRs are enriched in module vii. To further explore the connection between CR classes and network modules, we compute the mean shortest path between two given CR classes and evaluate the significance over the mean shortest path through permuting the graph while keeping the degree of each node. As a result, we get a network of CR-class interaction in different module. This network contains 10 nodes (CR-classes) and 16 edges, which appear in different modules. For example, the SWI/SNF complex is connected to histone acetylation in module vii and connected to INO80 complex in module v. These results may suggest that each of these modules possesses different biological functions.

We further explore the function of these modules by performing functional enrichment analysis on CRs’ target genes within each module. The results show that these modules have different biological functions. Module ii and iii are associated with embryonic developmental process; Module i, iv and vii are associated with development process of different tissues; Modules v and vi are important for nutrition supply (Fig. 7).

## Discussions and Conclusions

Differentiation from mESC to lineage specific tissues involves complicated interplay between epigenome and transcriptome. Chromatin factors serve as the important bridge to connect these two layers. After sensing the external signals, chromatin factors may modulate the chromatin state by re-organizing its structure, changing accessibility, reading, writing, or erasing the modifications. Eventually, those chromatin state changes will cause subsequent gene expression change. Due to its importance, increasing attention has been paid to CRs. ChIP-Seq technology has been extended to study CR’s interaction with chromatin, its genomic location, and combinatorial regulation. In this paper, we assess the regulatory roles from different viewpoint. We look into the gene expression data during mESC differentiation to model the relationships among CRs, TFs, and target genes. This information will tell us how CRs convey the regulatory signal from chromatin to expression level.

To this end, we proposed a new method, named MOCHA, to detect the chromatin modulation of transcriptional regulation by systematically integrating the transcriptional regulation information, chromatin modulation information, and phenotype information. We apply our method to study mouse embryonic stem cell differentiation. As a result, literature and database based validations suggest that MOCHA can recapitulate many known mechanism and infer novel mechanism during mESC differentiation. MOCHA may serve as a general tool in other studies.

Some limitations of MOCHA should be noted and additional efforts are required in further study. Some CRs, particularly enzymes, have relative high levels expression on different cell types but expression levels are stable across different conditions. Therefore, it’s hard to detect these CRs’ modulation from gene expression data in this case.

To assign the large amount of CR, TF, and TG triplets to a specific chromatin region, we used TG locations in genome to anchor the triplets. If the genome-wide CR and TF location data is available, we can integrate those data into our model as the further step^7^. This will help us reduce the false positives and better pinpoint the CR-TF-TG functional unit in a given genomic region.

## Methods and Materials

### Data collection

We compiled gene expression profiles from three distinct mESC lines (R1, J1 and V6.5) undergoing undirected differentiation into embryoid bodies (EBs) over a period of two weeks^35^. These data is downloaded from GEO (GSE2972, GSE3231, and GSE3749). Each dataset contains 11 time points and on each time points there are three replicates. We collected 943 TFs from animal TF database and collected 597 CRs in three categories (Supplementary dataset 1). The first category is epigenetic factors (Epi) including histone modification associated factors and DNA methylation associated factors. The second category is the chromatin modification enzymes (CME) including K-demethylase family enzymes, K-acetyltransferase family enzymes, and K-methyltransferase family enzymes^36^. The third category is ATP-dependent chromatin remodeling proteins (CRP) including SWI/SNF family members, CHD family members, ISWI family members, and INO80 family members.

### Transcriptional regulatory network reconstruction

The mutual information of TF (X) and a potential target gene (Y) is as follows:


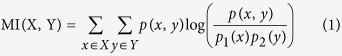


To compute the mutual information, we apply the fast calculation of pairwise mutual information method by using a Gaussian kernel estimator to estimate the distribution^37^. We apply t-test to tell whether the mutual information score is statistically significant.

### Modulational regulatory network reconstruction

In pluripotent embryonic stem cell, transcriptional regulation is controlled by an ‘active’ chromatin state, and it has been reported that this state can switch the transcriptional regulation pattern. In other words, it implies that both the strength and pattern of associations between two gene profiles may vary as the intrinsic chromatin-state changes. Chromatin states are often associated with the levels of chromatin regulators, such as chromatin remodeling factors, chromatin modifying enzymes, and some epigenetic factors. Modulation relationship between three gene can be detected by the condition correlation^38^,^39^. Liquid association^14^ is an elaborate method to detect the genome-wide co-expression dynamics from gene expression data. In order to calculate liquid association and it’s statistically significance fast in many possible triplets, we propose an approximation approach to calculate the liquid association and it’s statistical significance.

Given the expression data standardized to have mean 0 and variance 1, the liquid association for transcriptional factor (X), target gene (Y) and chromatin regulator (Z) is defined as follows: LA(X,Y|Z) = E(*g*′(Z)), where g(z) = E(XY|Z = z). In order to calculate liquid association and it’s statistical significance fast in many possible triplets, we propose an approximation approach as follows to calculate the liquid association and it’s statistical significance.


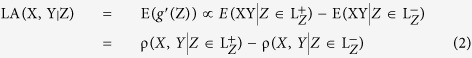


Where 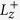 represents the set of samples on which gene Z is highly expressed. Similarly, the 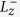 represents the set of samples on which gene Z is lowly expressed. ρ represents the Pearson correlation coefficient. On a null hypothesis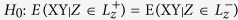, we apply a Fisher transformation based Z-test to test the significance of the LA.


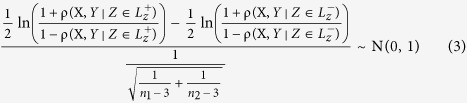


Where *n*_1_ represents the number of samples in which gene Z is highly expressed. Similarly, *n*_2_ represents the number of samples in which gene Z is lowly expressed. The statistical significance of the liquid association is often computed by permutation test, which generates as many as 10^5^ or 10^6^ artificial profiles *Z*′ of Z by randomly permuting data Z. This procedure is quite time consuming. Compared to this process, our method is fast in computation. In order to verify the efficiency of our method, we compare the p-values computed by the two methods on 10^5^ permutation data (Fig. 2(c)). The results show that the p-value computed by our method is consistent with the p-value computed by the permutation test.

### Reducing false positive rates

We can rank and predict a large number of triplets by MOCHA. However, there are many false positives in these triplets. In order to reduce the number of false positives, we designed a two-layer screening method. Firstly, we applied the Bonferroni adjustment to control the FDR. By cutting the q-value at 0.001, we filtered out some low confidence triplets. Secondly, we reduced the false positive rate at network level. Given the fact that chromatin regulators usually modulate transcription generally, we filtered out those triplets which have a chromatin regulator with low degree in the modulation network.

### Identifying key chromatin regulators and their modulation subnetwork

To further narrow down the number of CR, TF, TG triplets, we introduce a pluripotency index to incorporate the phenotype of the samples. The pluripotency index is defined as the mean expression of 16 core pluripotency genes (Pou5f1, Sox2, Nanog, Klf4, Myc, Tcf3, Esrrb, Zfp42, Nr0b1, Stat3, Sall4, Phc1, Klf2, Rest, Zfp281, and Tbx3). For each time point we can calculate this pluripotent index. This index allows us to monitor the phenotype change during the differentiation. Then we apply the Pearson correlation coefficient to measure the consistency between chromatin regulator/TF and phenotype.

All the four types of information can be evaluated by p-values. To give a modulation score for a given triplet, we apply Fisher’s combined probability test to integrate four p-values: mutual information p-value, liquid association p-value, the p-value of correlation between CR and phenotype, the p-value of correlation between TF and phenotype (Fig. 1). In this way, all the triplets have a modulation score (chi-square score) and an integrative p-value.


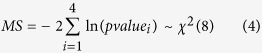


For each triplet, MOCHA assigns a modulation score (Chi-square statistics) and p-value. In this way, a chromatin regulation score is calculated for each chromatin regulator. For a given chromatin regulator, chromatin regulation score is defined as the sum of the modulation scores of the triplets which were modulated by this chromatin regulator. We can identify the key chromatin regulators by ranking the chromatin regulation score. To study a given chromatin regulator, we can extract its modulation subnetwork from the triplets which were modulated by this chromatin regulator.

### Inferring chromatin activity by Gaussian Bayesian network

Chromatin activity (CA) reflects the chromatin accessibility and modification via histone regulated by chromatin regulators. We assume that the chromatin activity is regulated by chromatin regulators. Transcriptional factors are trans-regulatory elements regulating the target genes under the condition that chromatin is accessible and active. Therefore, genes located in the same region are assumed to be regulated by their specific TFs and a common chromatin activity. We model the activity as a hidden variable and conditioned on the gene expression level of chromatin regulators. Then starting from the calculated triplets, this problem is deemed as parameter estimation from incomplete data in Bayesian network (Fig. 1). In a genomic region, regulations at two levels are considered in our model: One is the chromatin regulators’ regulation on chromatin activity; the other is the chromatin activity and TFs’ regulation on target genes. We assume that both level regulations are linear Gaussian. So the conditional probability distributions of three levels are as follows:


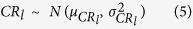



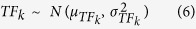



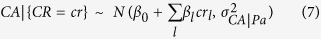



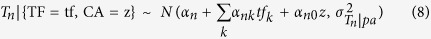


where *α*_*nk*_ is the regulatory strength of the *k*-th TF on the *n*-th target gene; *α*_*n*0_ is the regulatory strength of CA on the *n*-th target gene; *β*_*l*_ is the effect of *l*-th CR on chromatin activity. *α*_*n*_ and *β*_0_ are the constant terms of *T*_*n*_ and CA. The parameter is denoted by 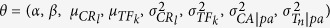.

Therefore, we can get the joint distribution:


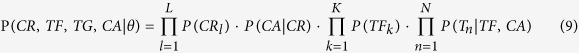


To estimate the parameters from the ESC differentiation data D, we use Maximum Likelihood Estimation (MLE) to maximize the likelihood function. Hard-assignment expectation maximization (EM) is used to maximize the log-likelihood function of the complete data.


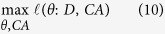


## Additional Information

**How to cite this article**: Duren, Z. and Wang, Y. A systematic method to identify modulation of transcriptional regulation via chromatin activity reveals transcription factor network during mESC differentiation. *Sci. Rep.* **6**, 22656; doi: 10.1038/srep22656 (2016).

## Supplementary Material

Supplementary Information

Supplementary Dataset 1

Supplementary Dataset 2

Supplementary Dataset 3

Supplementary Dataset 4
