# Supplementary material for: A systematic method to identify modulation of transcriptional regulation via chromatin activity reveals regulatory network during mESC differentiation
Source: Sci Rep. 2016 Mar 7;6:22656. doi: 10.1038/srep22656 (PMC4780077; doi:10.1038/srep22656)
Supplement: Supplementary Information [file srep22656-s1.pdf]

Supplementary Information

A systematic method to identify modulation of transcriptional regulation via chromatin activity reveals regulatory network during mESC differentiation

Zhana Duren and Yong Wang*

Academy of Mathematics and Systems Science, National Center for Mathematics and Interdisciplinary Sciences, Chinese Academy of Sciences, Beijing 100190, China

* Corresponding author:

Yong Wong, Email: ywang@amss.ac.cn

Supplementary dataset legends

Supplementary dataset 1. List of chromatin regulators.

Supplementary dataset 2. List of TF-target pairs with corresponding mutual information scores and p-values.

Supplementary dataset 3. List of CR-TF-TG triplets with mutual information, liquid association, phenotype and corresponding p-values.

Supplementary dataset 4. CR-CR network.

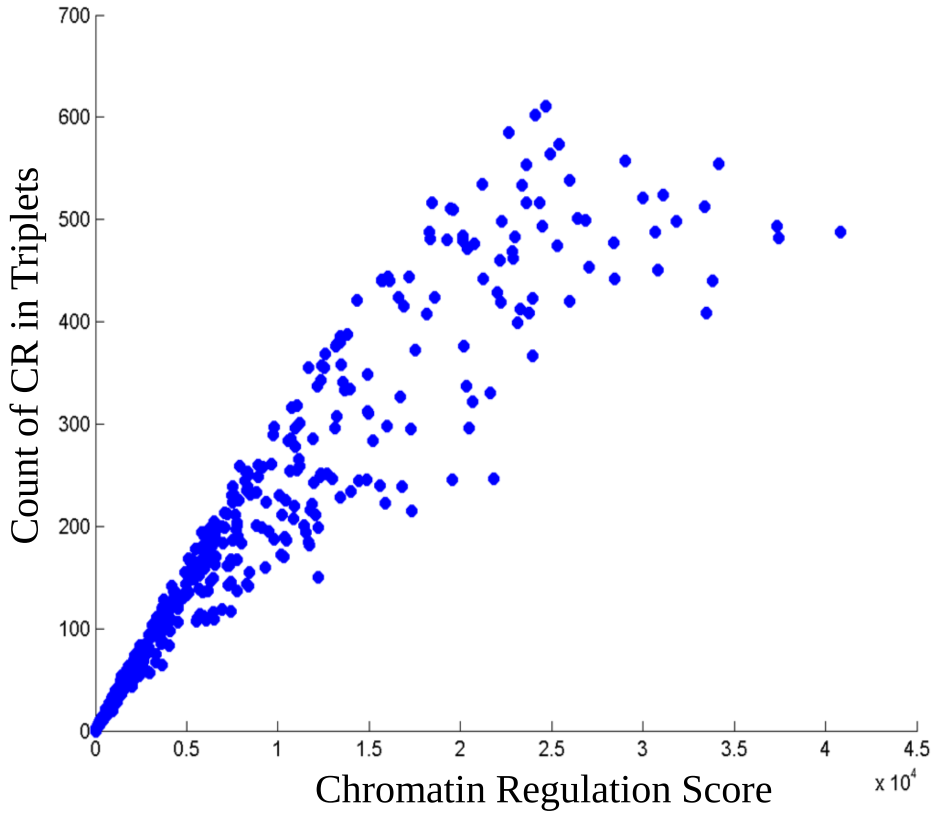

Supplementary Figure 1. Chromatin regulation score versus count of CR in triplets.

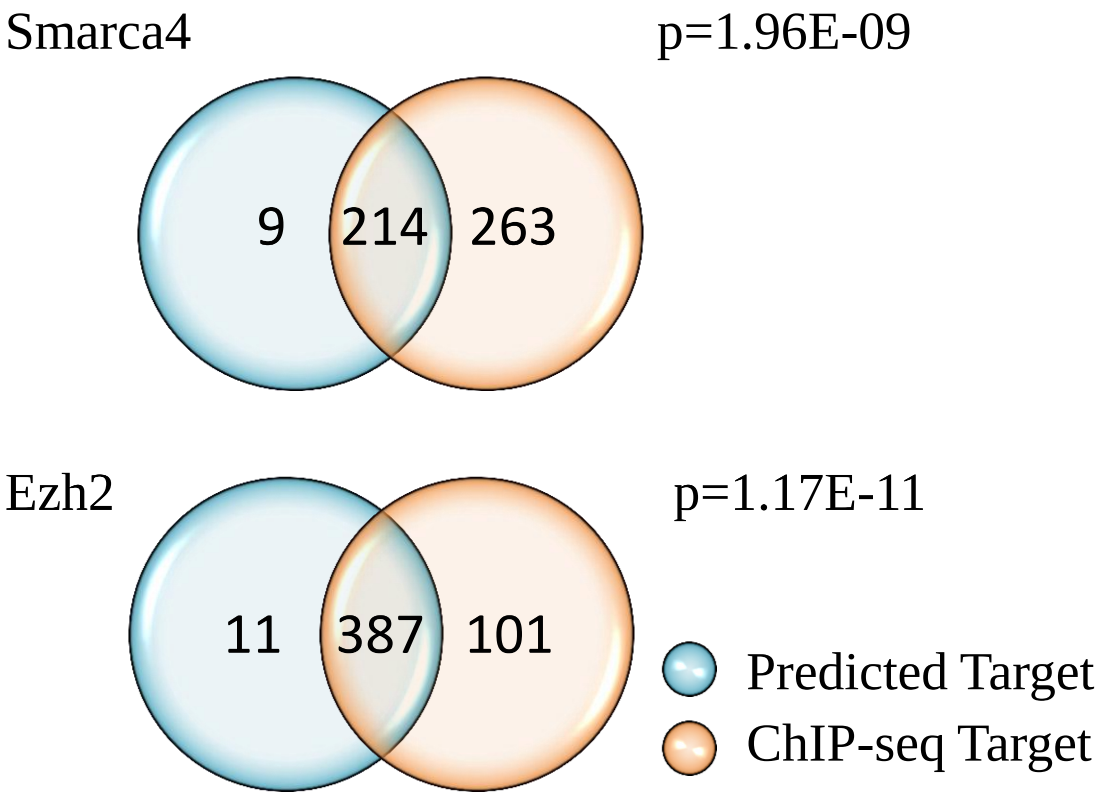

Supplementary Figure 2. Comparison of predicted CR target region and CRs' target region measured by ChIP-seq. We apply our method to predict target region on polycomb complex member Ezh2 and BAF complex member Smarca4 (BAF190) which have ChIP-seq data to compare in mESC. As a result, 214 out of 223 and 387 out of 498 target regions are validated by the Smarca4 and Ezh2 ChIP-seq data respectively. Both of these two overlapping are significant by hypergeometric test.

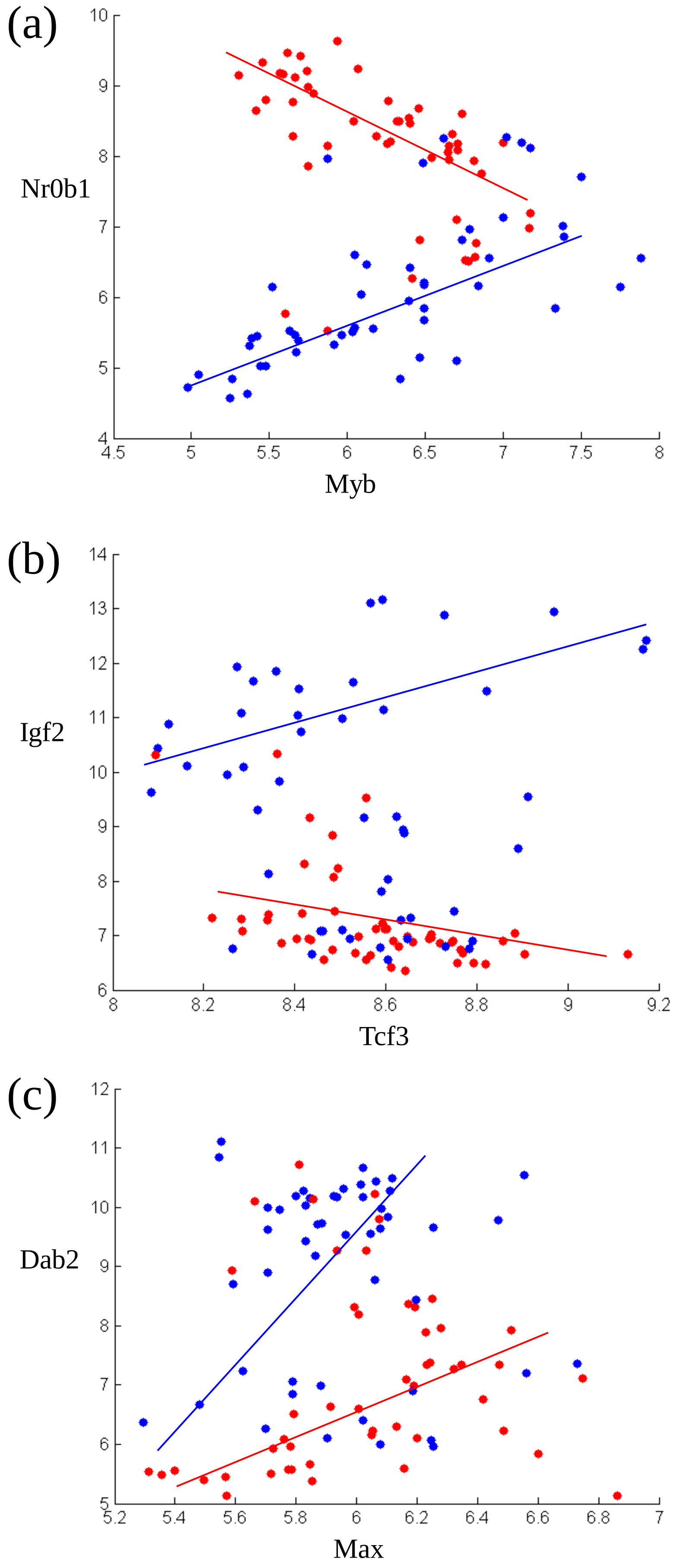

Supplementary Figure 3. Expression pattern of TF-TG changes dynamically during the stem cell differentiation. Red nodes represent the samples in pluripotent stage and blue nodes represent the samples in differentiated stage.
